# Supplementary material for: CLCFM3: A 3D Reconstruction Algorithm Based on Photogrammetry for High-Precision Whole Plant Sensing Using All-Around Images
Source: Sensors (Basel). 2025 Sep 18;25(18):5829. doi: 10.3390/s25185829 (PMC12473635; doi:10.3390/s25185829)
Supplement: Supplementary file 1 [file sensors-25-05829-s001.zip › sensors-3679136-supplementary.pdf]

Supplementary Materials:

Figure S1 shows the results of reconstruction of 3D point clouds of four soybean varieties using the proposed method.

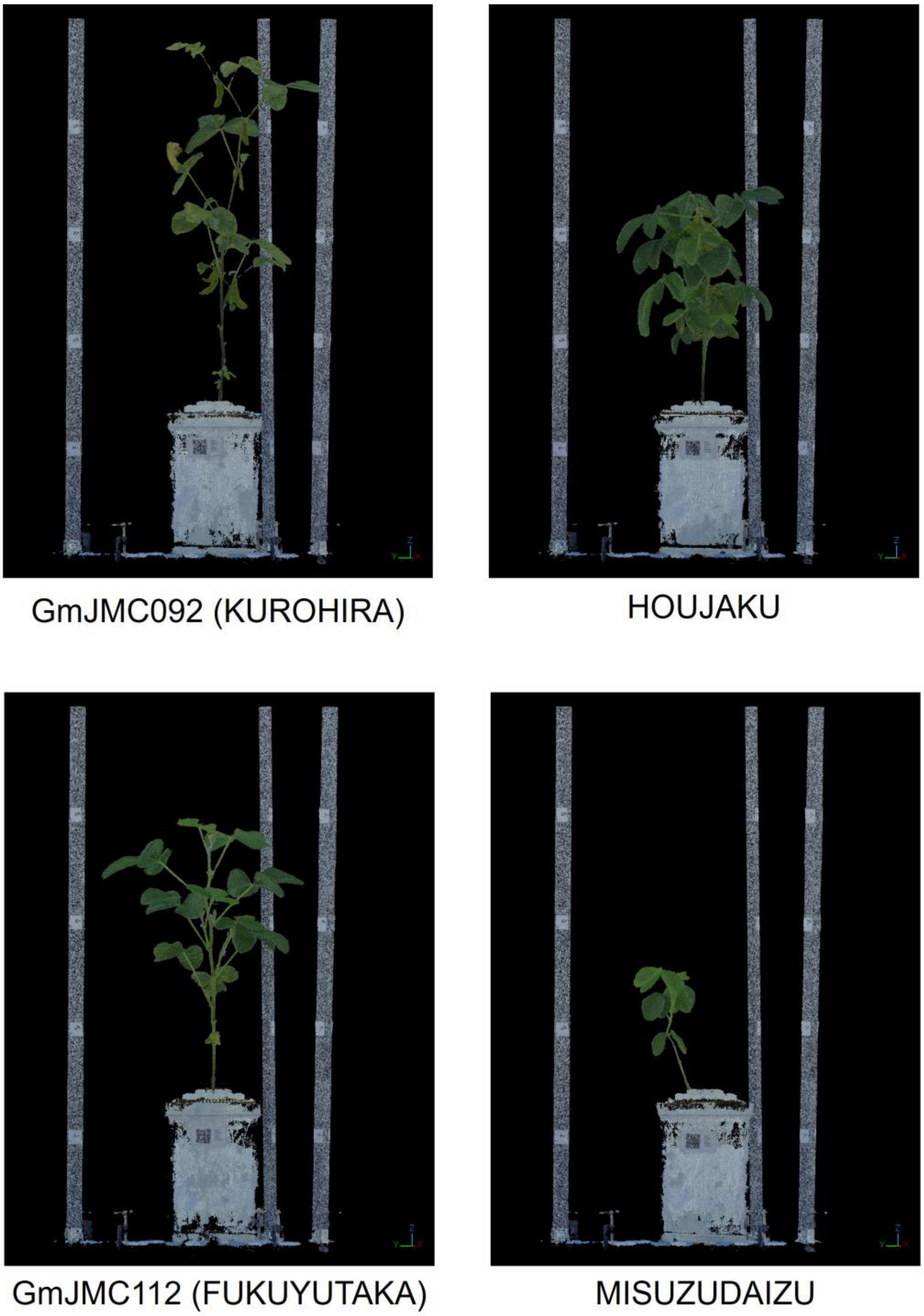

Figure S1. Results of reconstruction of 3D point clouds of four soybean varieties using the proposed method.

Figure S2 shows the results of reconstruction of 3D point clouds for the image in Fig. S1 using Metashape.

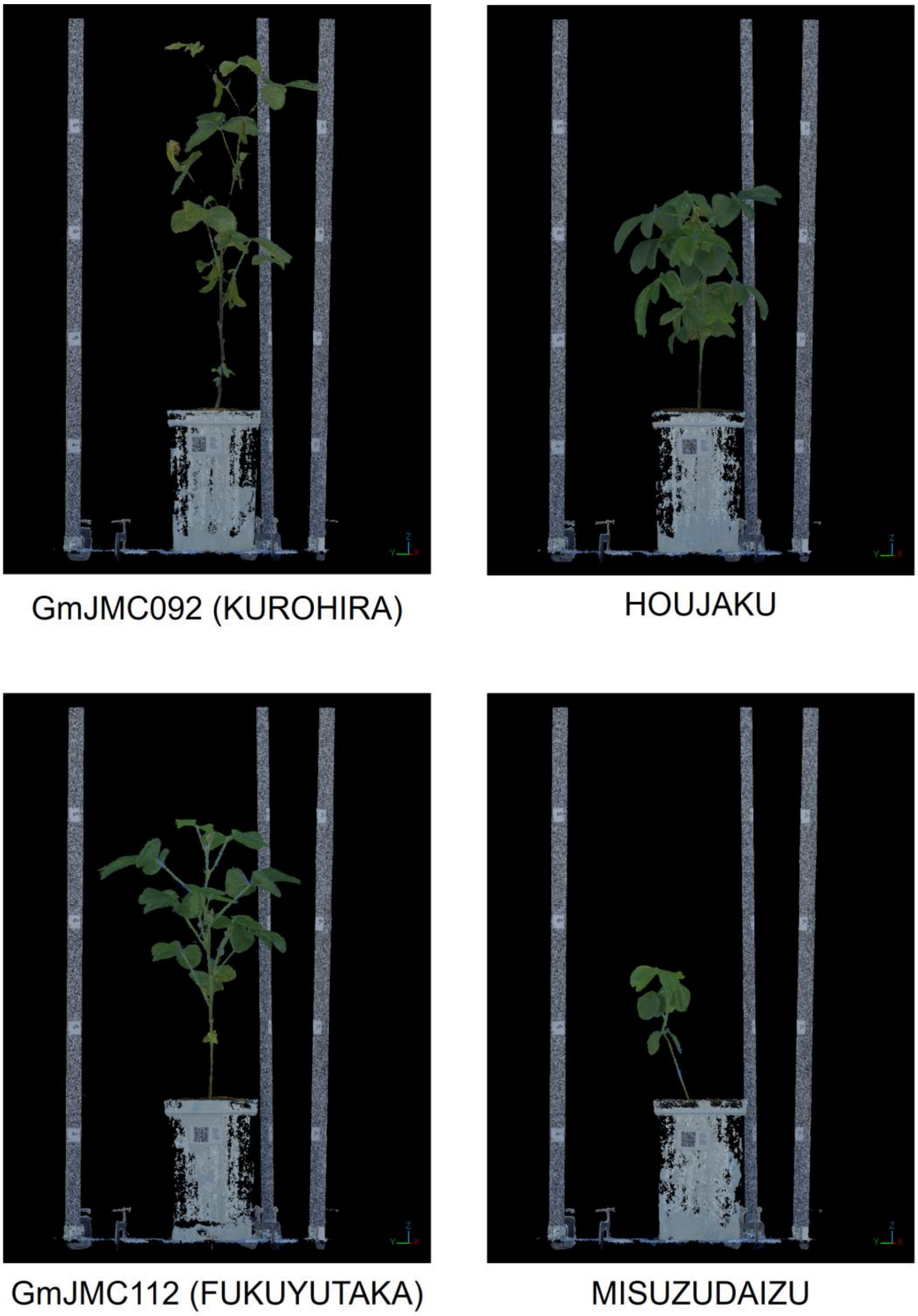

Figure S2. Results of reconstruction of 3D point clouds for the same image in Fig. S1 using Metashape.

Figure S3 shows the results of running the analysis in Fig. 5 using Metashape 1.6.

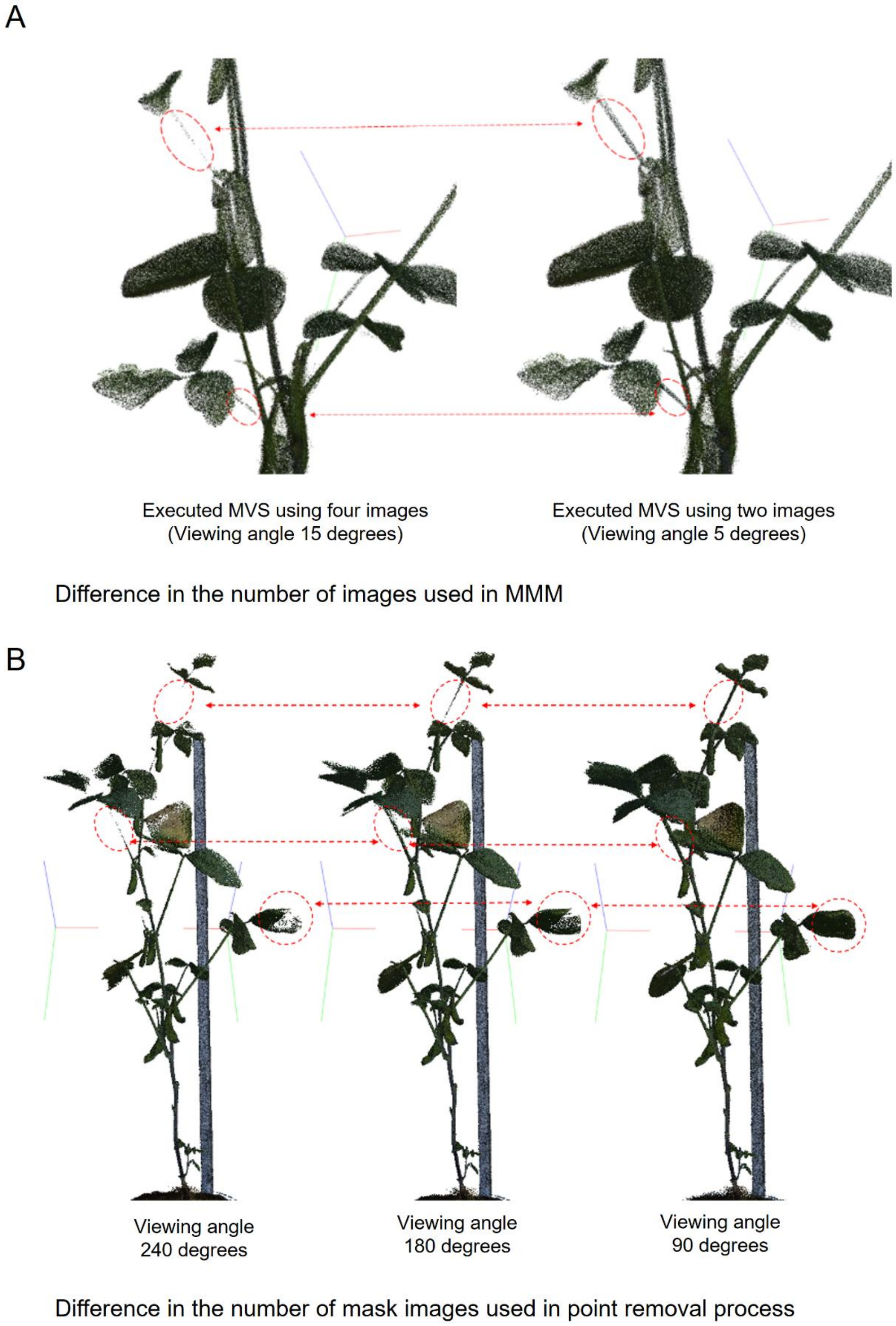

Figure S3. Results of running the analysis in Figs. 5 and 6 using Metashape 1.6.

Figure S4 shows the results of running the analysis in Fig. 6 using Metashape 1.6.

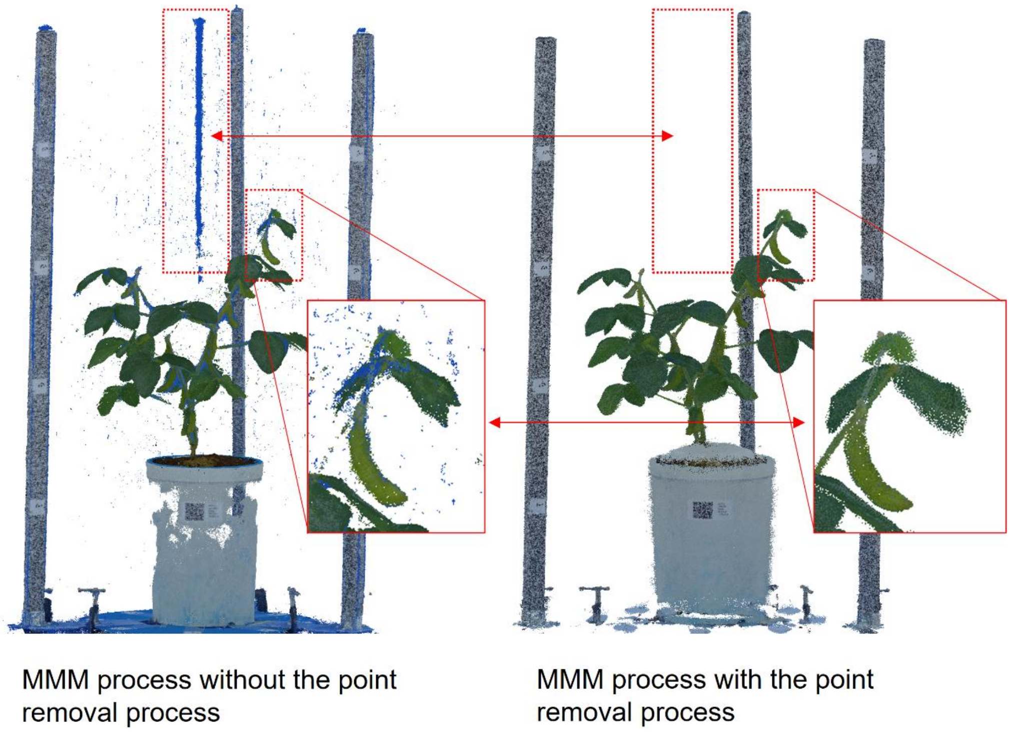

Figure S4. Results of running the analysis in Fig. 7 using Metashape 1.6.
